# Substituted Pyrazinecarboxamides as Abiotic Elicitors of Flavolignan Production in *Silybum marianum* (L.) Gaertn Cultures *in Vitro*

**DOI:** 10.3390/molecules15010331

**Published:** 2010-01-14

**Authors:** Lenka Tůmová, Jiří Tůma, Klara Megušar, Martin Doležal

**Affiliations:** 1 Faculty of Pharmacy in Hradec Králové, Charles University in Prague, Heyrovského 1203, 500 05 Hradec Králové, Czech Republic; E-Mails: martin.dolezal@faf.cuni.cz (M.D.); klara.megusar@sandoz.com (K.M.); 2 Department of Biology, Faculty of Education, University of Hradec Králové, Víta Nejedlého 573, 500 03 Hradec Králové, Czech Republic; E-Mail: jiri.tuma@uhk.cz (J.T.)

**Keywords:** pyrazinecarboxamides, abiotic elicitors, milk thistle (*Silybum marianum* L.), silymarin, flavonolignan production, callus culture

## Abstract

Substituted pyrazinecarboxamides markedly influenced production of flavonolignans in *Silybum marianum* callus and suspension cultures. In this study the effect of two compounds, *N*-(3-iodo-4-methylphenyl)pyrazine-2-carboxamide (**1**) and *N*-(3-iodo-4-methylphenyl)-5-*tert*-butyl-pyrazine-2-carboxamide (**2**), as abiotic elicitors on flavono-lignan production in callus culture of *S. marianum* was investigated. Silymarin complex compounds have hepatoprotective, anticancer and also hypocholesterolemic activity. *In vitro* flavonolignan concentration in cells is very low and the elicitation is one of the methods to increase production. Elicitors were tested at three concentrations and at different culture times. In the case of elicitation with **1**, the greatest increase of flavonolignan and taxifoline production was observed at concentration c_1a _after 6-hours of elicitation and after 24 and 72-hours at concentration c_1b_. However, increased production of silychristin, one of the compounds in the silymarin complex, was achieved after only 6-hours elicitation with c_1a_ (2.95 × 10^-4^ mol/L). The content of silychristin was 2-times higher compared to the control sample. An increased production of silychristin was reached with compound **2 **at the concentration c_2 _(2.53 × 10^-3^ mol/L) after 72 h of elicitation. The production of silychristin in this case was increased 12-times compared to control.

## 1. Introduction

Many polycyclic compounds of biological or industrial significance have the pyrazine ring in their structure. There is a high interest in these compounds due to the widespread occurrence of pyrazines in Nature, especially in the flavor compounds of many food systems, their effectiveness at very low concentrations, as well as the still increasing number of applications of synthetic pyrazines in the flavor, fragrance and pharmaceutical industries. In plants or insects, some pyrazines play roles as attractants, pheromones and signal substances, and similar substances are found in food [1].

Some ring substituted pyrazinecarboxamides, tested *in vitro*, showed high antimycobacterial activity. A simple pyrazine compound, 3-amino-6-chloropyrazine-6-carboxylic acid showed an anti auxin behavior [2,3]. The herbicidal and abiotic elicitation activity of novel pyrazine derivatives has been evaluated [2,4]. Substituted *N*-phenylpyrazine-2-carboxamides were already tested on callus culture *Ononis arvensis* L. All of them increased flavonoid production [5,6].

Silymarin from *Silybum marianum* is a phytomedicine traditionally used in the treatment of liver disorders and is now included as a complementary and alternative medicine for liver diseases. The active extract of *S. marianum*, known as silymarin, is a mixture of flavanolignans, namely silybin, silydianin, and silychristine. Although the whole plant is used as a medicinal, the seeds contain the highest content of silymarin (1.5–3.0%). Most of its hepatoprotective properties are attributed to the presence of silybin, which is the main constituent (60–70%) of silymarin. The mechanism of action of silymarin is not well understood. Reports indicate that it acts in multiple ways. Silymarin stabilizes the membrane structure of hepatocytes and thus prevents toxins from entering the cell through enterohepatic recirculation. It promotes liver regeneration by stimulating nucleolar polymerase A and by increasing ribosomal protein synthesis. Silymarin is one of the most successful examples of development of a modern drug from a traditional medicine. However, standardization of silymarin in its various formulations and effective dosages is still lacking [7]. Compounds of the silymarin complex have other interesting activities, e.g. anticancer and cancer protective and also hypocholesterolemic activity. These effects were demonstrated in a large variety of illnesses of different organs, e.g. prostate, lungs, CNS, kidneys, pancreas and also in skin protection. Proapoptotic activity of silybin in pre-and/or cancerogenic cells and anti-angiogenic activity of silybin are other important findings that bring silymarin preparations closer to application in the cancer treatment [8]. Silybin inhibits expression of HIF-1 alpha through suppression of protein translation in prostate cancer cells [9]. Kim determined the effect of silibinin on TNF-α-induced MMP-9 expression (tumor necrosis factor (TNF)-α) in gastrin cancer cells. [10] Silibinin possesses strong antioxidant activity and also modulates many molecular changes caused by xenobiotics and ultraviolet radiation to protect the skin [11]. 

Two ring-substituted (alkylated, brominated) phenylamides of pyrazinecarboxylic acid markedly influenced production of flavonolignans in callus and suspension culture of *S. marianum* [12]. Additionally the effects of foliar or soil fertilization of 5*-tert-*butyl-*N-m*-tolylpyrazine-2-carboxamide on the vegetative and reproductive growth, some physiological parameters, seed yield and silymarin content of field grown milk thistle (*S. marianum*) plants were studied and the accumulation of flavonoids and silymarin compounds in the seeds was positively influenced. Treatments with foliar fertilizer and 5*-tert-*butyl-*N-m*-tolylpyrazine-2-carboxamide resulted in improvement of plant biomass, nutrient accumulation, flowering rate and seed yield and quality [13,14]. 

The aim of this study was to evaluate the effects of other two compounds, *N*-(3-iodo-4-methyl-phenyl)pyrazine-2-carboxamide (**1**) and *N*-(3-iodo-4-methylphenyl)-5-*terc*-butylpyrazine-2-carbox-amide (**2**), primarily prepared and described as antimycobacterial compounds [15], as abiotic elicitors on flavonolignan production in callus culture of *S. marianum*.

## 2. Results and Discussion

**Figure 1:**
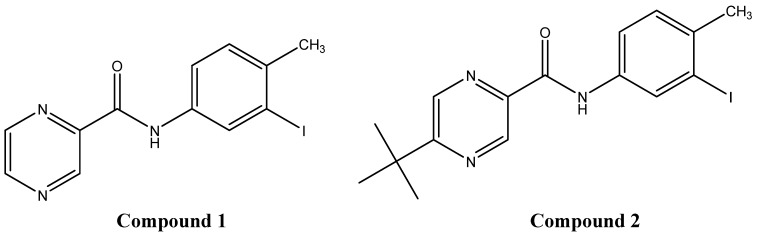
Structures of compounds **1** and **2**.

The condensation of chlorides of substituted pyrazinecarboxylic acids with ring substituted anilines yielded several substituted pyrazinecarboxylic acid amides [15]. Two newly synthesized substituted amides of pyrazinecarboxylic acid, *N*-(3-iodo-4-methylphenyl)pyrazine-2-carboxamide (**1**) and *N*-(3-iodo-4-methylphenyl)-5-*tert*-butylpyrazine-2-carboxamide (**2**), (Figure 1) were investigated as abiotic elicitors of flavonolignan production in callus culture of *S. marianum*. Both studied compounds were designed in preference with the lipophilic and/or electron-withdrawing substituents on the benzene moiety.

### 2.1. Elicitation with N-(3-iodo-4-methylphenyl)pyrazine-2-carboxamide (**1**)

**Figure 2:**
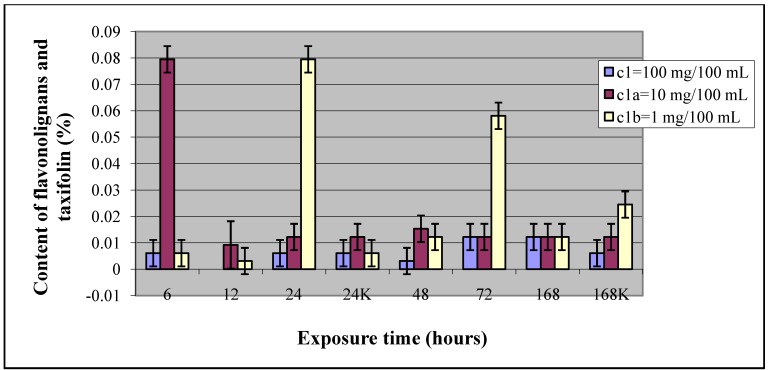
Dependence of the content of flavonolignans and taxifolin in callus culture *Silybum marianum* related to the elicitor exposure times compared between treatments with three different concentrations of compound **1** and between controls.

Compound **1** at concentration c_1_ (Table 1) had no important effect on *S. marianum* calluses, since there is no statistically significant increase or decrease of flavonolignan and taxifolin production. As shown in the Figure 2 there are two decreases in flavonolignan and taxifolin production, after 12 and 48-hours elicitation, and two increases, after 72 and 168-hours elicitation. However, these results are not statistically significant and therefore is was not appropriate to make any conclusions from this fact.

The effect of compound **1** at concentrations c_1a_ and c_1b_ was greater. There was one statistically significant increase in flavonolignan and taxifolin production with c_1a_, after 6-hours elicitation (7-times control value), and two with c_1b_, after 24 (13-times control value) and 72-hours (10-times control value) elicitation. The detailed HPLC analysis shows that in case of 6-hours’ elicitation with compound **1** at concentration c_1a_ half of the detected content corresponded to taxifolin (0.04%), while the other half was the flavonolignan silychristin (0.04%), whereas after 24 and 72-hours of elicitation with concentration c_1b_ almost the whole detected content was attributed to taxifolin. Since the uses of *S. marianum* in therapy are based on the pharmacological activities of silymarin, a component of which is also silychristin, the important effect of elicitation with compound **1** in our study is after 6-hours with concentration c_1a _= 10 mg/100 mL. Moreover, with concentration c_1b_ after 168-hours elicitation there is a decrease in content of flavonolignans and taxifolin, however, the decrease is not statistically significant.

**Table 1:** Flavonolignans and taxifolin content (%) in callus culture *S. marianum* after elicitation with compound **1**.

Elicitor concentration (mg/100 mL)	Exposure time (hours)	Content of flavonolignans and taxifolin (%)	SD	Statistic value - *t*
	6	0.006	0.005	0.00
****	12	0.000	0.000	1.63
	24	0.006	0.005	0.00
**100 (c_1_)**	**24K**	**0.006**	0.005	-
	48	0.003	0.005	0.577
	72	0.012	0.005	1.16
	168	0.012	0.005	1.16
	**168K**	**0.006**	0.005	-
	6	0.080	0.005	12.7
****	12	0.009	0.009	0.408
	24	0,012	0.005	0,00
**10 (c_1a_)**	**24K**	**0.012**	0.005	-
	48	0.015	0.005	0.577
	72	0.012	0.005	0.00
	168	0.012	0.005	0.00
	**168K**	**0.012**	0.005	-
	6	0.006	0.005	0.00
****	12	0.003	0.005	0.577
	24	0.080	0.005	13.9
**1 (c_1b_)**	**24K**	**0.006**	0.005	-
	48	0.012	0.005	1.16
	72	0.058	0.005	9.82
	168	0.012	0.005	231
	**168K**	**0.025**	0.005	-

**K**-control (without elicitor treatment).

In Figure 2 the dependence of the content of flavonolignans and taxifolin related to the elicitor exposure times compared between different concentrations of compound **1** and between controls is presented. All experimental analyses were carried out in a minimum of three independent samples for each elicitation period and each concentration of elicitor.

### 2.2. Elicitation with N-(3-iodo-4-methylphenyl)-5-tert-butylpyrazine-2-carboxamide (**2**)

**Figure 3:**
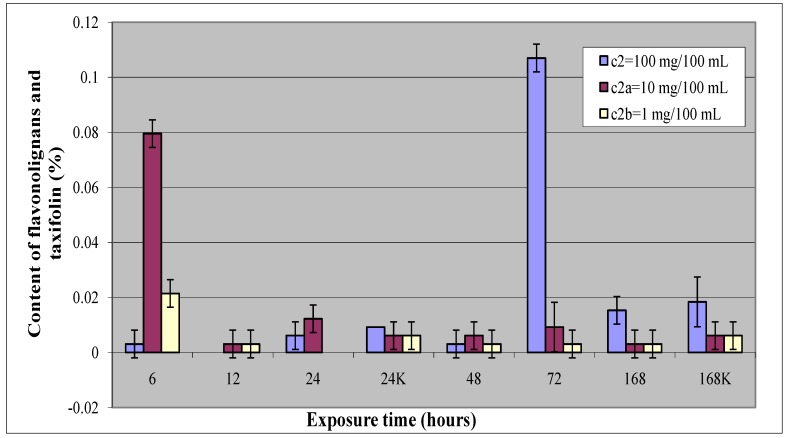
Dependence of the of flavonolignans and taxifolin content in callus culture *Silybum marianum* related to the elicitor exposure compared between treatments with three different concentrations of compound **2** and between controls. All experimental analyses were carried out in a minimum of three independent samples for each elicitation period and each concentration of elicitor.

Results listed in Table 2 show one statistically significant increase in flavonolignan and taxifolin production at each concentration of compound **2**; after 72-hours elicitation at c_2_ (12-times control value) and after 6-hours elicitation at c_2a_ (13-times control value) and c_2b_ (4-times control value). The detailed HPLC analysis of these three results shows that with the c_2_ concentration the main detected compound was the flavonolignan silychristin, whereas with c_2a_ and c_2b_ concentrations only taxifolin was detected. As shown in Figure 3, declines in the flavonolignans and taxifolin contents were observed. These effects were found after 6, 12 and 72-hours elicitor treatment at concentration c_2_, and after 24-hours elicitor treatment at concentration c_2b_. An increase in flavonolignan and taxifolin production after 24-hours elicitation at concentration c_2a_ was noted.

**Table 2:** Flavonolignans and taxifolin content (%) in callus culture *S. marianum* L. after elicitation with compound **2**.

Elicitor concentration (mg/100 mL)	Exposure time (hours)	Content of flavonolignans and taxifolin (%)	SD	Statistical value - *t*
	6	0.003	0.005	1.63
****	12	0.000	0.000	-
	24	0.006	0.005	0.816
**100 (c_2_)**	**24K**	**0.009**	0.000	-
	48	0.003	0.005	1.63
	72	0.107	0.005	26.1
	168	0.015	0.005	0.408
	**168K**	**0.018**	0.009	-
	6	0.080	0.005	13.9
****	12	0.003	0.005	0.577
	24	0.012	0.005	1.16
**10 (c_2a_)**	**24K**	**0.006**	0.005	-
	48	0.006	0.005	0.00
	72	0.009	0.009	0.408
	168	0.003	0.005	0.577
	**168K**	**0.006**	0.005	-
	6	0.021	0.005	2.89
****	12	0.003	0.005	0.577
	24	0,000	0.000	1.63
**1 (c_2b_)**	**24K**	**0.006**	0.005	-
	48	0.003	0.005	0.577
	72	0.003	0.005	0.577
	168	0.003	0.005	0.577
	**168K**	**0.006**	0.005	-

**K**-control (without elicitor treatment).

Results mentioned above are also shown in Figure 3, which shows the effect of elicitation after different exposure times in comparison between three different concentrations of compound **2**.

An increased flavonolignan production in *S. marianum* callus and suspension cultures after treatment with similar compounds, *i.e*., the 3-methylamide of 5-*tert*-butylpyrazine-2-carboxylic acid and the 5-bromo-2-hydroxyphenylamide of 5 *tert*-butyl-6-chloropyrazine-2-carboxylic acid were also observed in our previous studies [12]. The production of flavonolignans in *S. marianum* callus cultures was generally very low. To clarify this phenomenon we propose the following scenario. The used callus cultures were old (72^nd^–74^th^ passages) and therefore the production of secondary metabolites was to poor. It could be possible that ethanol which was used as a solvent for elicitors and was also added to control samples, caused poorer production of examined secondary metabolites. Preferable for this experiment would be the application of suspension cultures.

Suspension cultures are morphologically more homogenous than callus tissue, thus, likely to show a more uniform biochemical response to elicitor action than the callus tissue. Homogeneous contact between cells in the callus and the culture medium is lacking, making reproducibility of experiments extremely difficult if the size or thickness of callus is slightly changed. 

## 3. Experimental

### 3.1. Abiotic elicitors in Silybum marianum L. callus culture

A study was made about effect of several compounds on the production of silymarin in *S. marianum* cultures. Yeast extract stimulated the accumulation and production of silymarin, chitin and chitosan stimulated only silymarin accumulation. Furthermore, jasmonic acid potentiated the yeast extract effect, and methyljasmonate strongly promoted the accumulation of silymarin by acting on number of steps of the metabolic pathway. [12] There was testing the effect of two substituted amides of pyrazinecarboxylic acid and the results showed that they markedly influenced production of flavonolignans in an *in vitro* culture. [8] 

### 3.2. Material and methods

Callus culture was derived from the germinating seeds of plant *Silybum marianum* (L.) Gaertn. (*Asteraceae*). Calluses were cultivated on MS (Murashige and Skoog) medium [16] containing NAA (naphtaleneacetic acid) as growth regulator at a concentration of 5.4 × 10^-5^ mol/L. Callus cultures were cultivated on paper bridges in Erlenmeyer flasks. These cultures were incubated in growth chambers at laboratory temperature under a 16 hours photoperiod. As elicitors compounds **1** (*N*-(3-iodo-4-methyl-phenyl)pyrazine-2-carboxamide, C_12_H_10_IN_3_O, m.w. 339.1) and **2** (5-*tert*-butyl-*N*-(3-iodo-4-methyl-phenyl)pyrazine-2-carboxamide, C_16_H_18_IN_3_O, m.w. 395.3) each at three different concentrations (c_1_, c_1a_, c_1b_ for **1**, and c_2_, c_2a_, c_2b_ for **2**) were used:

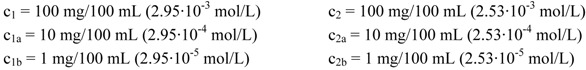


Elicitation of callus cultures was carried out the 21^st^ day after last passage. For each experiment, this means one concentration of one elicitor, 32 flasks were used. In 24 flasks a certain concentration of elicitor (1 mL) was pipetted into the nutrient medium, the last 8 flasks were used as control (**K**) samples into which 96% ethanol (1 mL) was pipetted. Every 6, 12, 24, 48, 72 and 168 hours we withdrew the calluses, controls were withdrawn after 24 and 168 hours. At each time interval and concentration we withdrew four flasks. After withdrawal, calluses were dried on filter paper at laboratory temperature (25 °C) and pulverized, weighed and used for determination of flavonolignans.

### 3.3. Flavonolignan analysis

Flavonolignan analysis was done by high-performance liquid chromatography (HPLC). A sample of of callus tissues (0.100 g, dry mass) was extracted twice (in a water bath under reflux condenser) with 80 % V/V aqueous methanol (10 mL) for 10 min. The extract was filtrated and then concentrated to 10 mL by vacuum distillation. Approximately 1.7 mL of distillate was placed in vials (after filtration through a 0.45 µm microfilter) and injected into the HPLC system.

HPLC analysis was performed on a Jasco chromatography system (pump PU-2089, detector MD-2015, autosampler AS-2055) equipped with a LiChrospher RP-18 250x4 (5 μm) column. Detection was performed by diode array detector with a wave-length span from 190 – 450 nm. The wave-length at 288 nm was used. Volume of injection was 20 µL. Mobile phase was composed of two phases: phase A – methanol solution of *o*-phosphoric acid (0.15% W/V), and phase B—Water solution of *o*-phosphoric acid (0.15% W/V). The elution profile was changing through time. At first elution was linear gradient with 100% of phase B at time t = 0 min and 50% of each phase (V/V) at time t = 5 min. Then elution was isocratic with 50% of each phase (V/V) until t = 25 min. The flow was 1.4 mL/min. As standards silymarin and taxifolin were used. [12] To determine whether there was difference between values of samples one–way analysis of variance (ANOVA) was applied. Values of p ≤ 0.05 were considered as significantly different. The differences between means were determined using Turkey´s multiple comparison test.

## 4. Conclusions

In the case of elicitation with compound **1** (*N*-(3-iodo-4-methylphenyl)pyrazine-2-carboxamide) on callus cultures of *S. marianum* the greatest increase of flavonolignan and taxifolin production was observed after 6-hours of elicitation at concentration c_1a_ and 24 and 72-hours of elicitation at concentration c_1b_. However, the increased production of silychristin, one of the compounds in silymarin complex, was reached only after 6-hours elicitation at c_1a _= 10 mg/100 mL (2.95 × 10^-4^ mol/L). The content of silychristin was 3-times higher compared to control sample. The elicitation with compound **2** (*N*-(3-iodo-4-methylphenyl)-5-*tert*-butylpyrazine-2-carboxamide) on callus cultures of *S. marianum* was higher after 72-hours exposure time at c_2_ and after 6-hours at c_2a_ and c_2b _concentrations. The increased production of silychristin was reached only after 72-hours elicitation at concentration c_2 _= 100 mg/100 mL (2.53 × 10^-3^ mol/L). The production of silychristin was increased 12-times compared to control sample. Achieved results will be the subject of future studies?
